# Supplementary material for: Content-rich biological network constructed by mining PubMed abstracts
Source: BMC Bioinformatics. 2004 Oct 8;5:147. doi: 10.1186/1471-2105-5-147 (PMC528731; doi:10.1186/1471-2105-5-147)
Supplement: Additional File 2 — The original results of the above study (non-essential files are deleted to keep the file size under the limit set by BMC bioinformatics). [file 1471-2105-5-147-S2.bz2 › chilibotAdditionalFile2/dip30/287ID1326789E1115/html/MAP2K1_RAF1.html]

 


 **MAP2K1** and **RAF1** 
  
Found 1002 abstracts in PubMed, retrieved 30.  
 

 What does Google say? 
 PDF only 
| .edu only 

---

**Interactive relationship** (e.g. stimulation, inhibition, etc)

**Inhibitory relationship**- BACKGROUND Raf  [ **RAF1** ]  kinase inhibitor protein RKIP, an inhibitor of Raf  [ **RAF1** ]  mediated activation of mitogen activated protein extracellular signal regulated kinase kinase MEK  [ **MAP2K1** ] , is expressed at lower levels in human C4 2B metastatic prostate cancer cells than in the parental non metastatic LNCaP prostate cancer cells from which they were derived.  Ref: 12813171 J Natl Cancer Inst, 2003
- These results show that raf 1  [ **RAF1** ]  induction suppresses neuroendocrine marker and hormone production in human gastrointestinal carcinoid cells via a pathway dependent on MEK  [ **MAP2K1** ]  activation.  Ref: 12851216 Am J Physiol Gastrointest Liver Physiol, 2003
- These results, along with others obtained using respiratory chain inhibitors and respiration deficient cells, lead to the conclusion that PD98059, while effectively inhibiting the peroxynitrite induced Raf  [ **RAF1** ]  MEK  [ **MAP2K1** ]  signaling leading to ERK1 2 phosphorylation, promotes an enforced mitochondrial formation of ROS inducing ERK1 2 phosphorylation via a Raf 1  [ **RAF1** ]  independent MEK  [ **MAP2K1** ]  dependent mechanism.  Ref: 12860392 FEBS LettFEBS Lett, 2003
**Neutral relationship**- Activation of raf 1  [ **RAF1** ]  in BON raf  [ **RAF1** ]  cells led to a marked induction of phosphorylated MEK  [ **MAP2K1** ]  and ERK1 2 within 48 h.  Ref: 12851216 Am J Physiol Gastrointest Liver Physiol, 2003
- We propose that FAK Src dependent, PAK1 mediated phosphorylation of MEK1  [ **MAP2K1** ]  on S298 is central to the organization and localization of active Raf  [ **RAF1** ]  MEK1  [ **MAP2K1** ]  MAPK signaling complexes,.  Ref: 12876277 J Cell BiolJ Cell Biol, 2003
- In contrast, VEGF activated Raf 1  [ **RAF1** ]  via Src kinase, leading to phosphorylation of tyrosines 340 and 341 and MEK1  [ **MAP2K1** ]  dependent protection from extrinsic mediated apoptosis.  Ref: 12843393 Science, 2003
- bFGF activated Raf 1  [ **RAF1** ]  via p21 activated protein kinase 1 PAK 1 phosphorylation of serines 338 and 339, resulting in Raf 1  [ **RAF1** ]  mitochondrial translocation and endothelial cell protection from the intrinsic pathway of apoptosis, independent of the mitogen activated protein kinase kinase 1  [ **MAP2K1** ]  MEK1  [ **MAP2K1** ] .  Ref: 12843393 Science, 2003
- Furthermore, treatment of BON raf  [ **RAF1** ]  cells with MEK  [ **MAP2K1** ]  inhibitors PD 98059 and U 0126 blocked raf 1  [ **RAF1** ]  mediated morphological changes and hormone suppression but not ERK1 2 phosphorylation.  Ref: 12851216 Am J Physiol Gastrointest Liver Physiol, 2003

**Non-interactive relationship** (e.g. studied together, co-existance, homology, etc.)

- Wild type Bcr, but not Bcr mutants defective in binding to AF 6, interferes with the Ras dependent stimulation of the Raf  [ **RAF1** ]  MEK  [ **MAP2K1** ]  ERK pathway.  Ref: 12808105 Mol Cell Biol, 2003
- Opposing roles of Ras Raf  [ **RAF1** ]  oncogenes and the MEK1  [ **MAP2K1** ]  ERK signaling module in regulation of expression and adhesive function of surface transglutaminase.  Ref: 12832399 J Biol Chem< MedlineTA>J Biol Chem, 2003
- The Ras Raf  [ **RAF1** ]  Mitogen activated protein kinase ERK kinase MEK  [ **MAP2K1** ]  extracellular signal regulated kinase ERK cascade couples signals from cell surface receptors to transcription factors, which regulate gene expression.  Ref: 12835716 Leukemia, 2003
- The ability of thrombin to repress Bim expression, and to protect cells from apoptosis, was reversed by U0126 or LY294002, suggesting that both the Raf  [ **RAF1** ]  > ; MEK  [ **MAP2K1** ]  > ; ERK1 2 and PI3K pathways cooperate to repress Bim and promote cell survival.  Ref: 12844349 Biochem J< MedlineTA>Biochem J, 2003
- In conclusion, activation of the Ras Raf  [ **RAF1** ]  MEK  [ **MAP2K1** ]  MAPK pathway through Shc mediates RET PTC induced thyroid cell dedifferentiation.  Ref: 12853977 Oncogene, 2003
- Inhibitors of the PI 3 kinase pathway attenuated Ang 1 induced ERK1 2 phosphorylation at a level up stream from Raf  [ **RAF1** ]  and MEK1  [ **MAP2K1** ]  2, but these inhibitors augmented Ang 1 induced p38 phosphorylation.  Ref: 12824293 FASEB J< MedlineTA>FASEB J, 2003
- This review will summarize our current understanding of the Ras Raf  [ **RAF1** ]  MEK  [ **MAP2K1** ]  ERK signal transduction pathway and the downstream transcription factors.  Ref: 12835716 Leukemia, 2003
- Signal transduction mediated by the Ras Raf  [ **RAF1** ]  MEK  [ **MAP2K1** ]  ERK pathway from cytokine receptors to transcription factors potential targeting for therapeutic intervention.  Ref: 12835716 Leukemia, 2003
- There was no activation of the Ras Raf  [ **RAF1** ]  MEK  [ **MAP2K1** ]  ERK pathway in the RhoA transfected cell line.  Ref: 12816757 Am J Physiol Gastrointest Liver Physiol< MedlineTA>Am J Physiol Gastrointest Liver Physiol, 2003
- Contrasting effects of Ras Raf  [ **RAF1** ]  oncogenes and their immediate downstream signaling module, MEK1  [ **MAP2K1** ]  ERK, on tTG expression, are consistent with adhesive function of surface tTG.  Ref: 12832399 J Biol Chem< MedlineTA>J Biol Chem, 2003
- Cotransfection assays demonstrated that TGF beta1 stimulation of IL 6 results from the synergistic collaboration of the Smad2, p38 NF kappaB, JNK c Jun AP 1, or Ras Raf  [ **RAF1** ]  MEK1  [ **MAP2K1** ]  cascades.  Ref: 12853969 Oncogene, 2003
- These events were accompanied by down regulation of the Raf 1  [ **RAF1** ]  MEK  [ **MAP2K1** ]  ERK pathway as well as diminished expression of Bcr Abl and cyclin D1, cleavage of p21 CIP1 and pRb, and induction of the stress related kinases JNK and p38 MAPK.  Ref: 12893773 BloodBlood, 2003
- Furthermore, additional signal transduction pathways interact with the Raf  [ **RAF1** ]  MEK  [ **MAP2K1** ]  ERK pathway to regulate positively or negatively its activity, or to alter the phosphorylation status of downstream targets.  Ref: 12835716 Leukemia, 2003
- Neuronal death and phosphorylation of components in this cascade were inhibited by the Raf  [ **RAF1** ]  inhibitor SB 386023, by the MEK  [ **MAP2K1** ]  inhibitor U0126, or by the MSK1 inhibitors H89 and Ro318220.  Ref: 12807421 J Neurochem< MedlineTA>J Neurochem, 2003
- Furthermore, we did not detect any alterations in the binding of paxillin to a number of focal adhesion proteins following either activation of the Raf  [ **RAF1** ]  >MEK  [ **MAP2K1** ]  >ERK pathway or expression of the S126A S130A form of paxillin in mammalian cells.  Ref: 12837288 Exp Cell Res, 2003
- Raf  [ **RAF1** ]  mitogen activated protein extracellular signal regulated kinase kinase MEK  [ **MAP2K1** ]  extracellular signal regulated kinase and MKK  [ **MAP2K1** ]  p38 mitogen activated protein kinase pathways play an important role in cellular survival and apoptosis.  Ref: 12839928 Cancer Res< MedlineTA>Cancer Res, 2003
- The present study demonstrates that SB 203580 activates members of the ERK cascade, c Raf  [ **RAF1** ] , MEK  [ **MAP2K1** ] , and ERK, in human monocytic THP 1 cells.  Ref: 12801911 J Biochem Tokyo , 2003
- These studies suggest that the ability of Ras and BCR ABL to activate NF kB involves an uncharacterized pathway which does not involve classic IKK activity and which suppresses the TNF induced IKK pathway through a Raf  [ **RAF1** ]  MEK  [ **MAP2K1** ]  Erk independent mechanism.  Ref: 12851413 J Biol Chem< MedlineTA>J Biol Chem, 2003
- NAMI A inhibits the PMA induced ODC gene expression in ECV304 cells Involvement of PKC Raf  [ **RAF1** ]  Mek  [ **MAP2K1** ]  ERK signalling pathway.  Ref: 12851698 Int J Oncol, 2003
- The phosphorylation of ERK1 2 could be blocked with a specific inhibitor of mitogen activated protein ERK kinase MEK  [ **MAP2K1** ] , suggesting that it was mediated through the highly conserved Ras Raf  [ **RAF1** ]  MEK  [ **MAP2K1** ]  ERK1 2 pathway.  Ref: 12807428 J Neurochem, 2003
- From this we conclude that a high affinity chitin oligosaccharide receptor system exists which links to the Raf  [ **RAF1** ] , MEK  [ **MAP2K1** ]  and ERK pathway in zebrafish cells.  Ref: 12881410 GlycobiologyGlycobiology, 2003
- The inhibition of NF kB is not observed in oncogenic Raf  [ **RAF1** ]  expressing cells and is not fully restored by the suppression of PI3 kinase PI3K or MEK  [ **MAP2K1** ]  pathways.  Ref: 12851413 J Biol Chem< MedlineTA>J Biol Chem, 2003
- These data indicate that paxillin is a target for phosphorylation downstream of the Ras activated Raf  [ **RAF1** ]  >MEK  [ **MAP2K1** ]  pathway.  Ref: 12837288 Exp Cell Res, 2003
- The Raf  [ **RAF1** ]  MEK  [ **MAP2K1** ]  inhibitor PD98059 enhances ERK1 2 phosphorylation mediated by peroxynitrite via enforced mitochondrial formation of reactive oxygen species.  Ref: 12860392 FEBS LettFEBS Lett, 2003
- Finally, specific inhibitors of the Ras Raf  [ **RAF1** ]  MEK  [ **MAP2K1** ]  ERK pathway inhibit LSF modification in vivo.  Ref: 12858339 J Cell Biochem, 2003
- Importantly, this form of cell death was inhibited in hippocampal neurones cultured from MSK1 mice and inhibitors of Raf  [ **RAF1** ]  or MEK  [ **MAP2K1** ]  had no additive neuroprotective effect.  Ref: 12807421 J Neurochem< MedlineTA>J Neurochem, 2003
- Inhibitors of Ras, Raf  [ **RAF1** ] , MEK  [ **MAP2K1** ]  and some downstream targets have been developed and many are currently in clinical trials.  Ref: 12835716 Leukemia, 2003
- The RAF  [ **RAF1** ]  MEK  [ **MAP2K1** ]  ERK MAPK signal transduction cascade is an important mediator of a number of cellular fates including growth, proliferation and survival.  Ref: 12879021 Oncogene, 2003
- It was also found that the Ras Raf  [ **RAF1** ]  MEK1  [ **MAP2K1** ]  cascade is activated by TGF beta1 and participates in the TGF beta1 induction of IL 6 in an AP 1 dependent manner.  Ref: 12853969 Oncogene, 2003
- Ras induced serine phosphorylation of the focal adhesion protein paxillin is mediated by the Raf  [ **RAF1** ]  >MEK  [ **MAP2K1** ]  >ERK pathway.  Ref: 12837288 Exp Cell Res, 2003
- Co treating PC 3 cells with quercetin significantly attenuated EGF and TGF alpha induced growth and phosphorylation of ErbB 2, ErbB 3, c Raf  [ **RAF1** ] , MAPK kinase 1 2 MEK1  [ **MAP2K1** ]  2, MAPK, Elk 1 and Akt 1.  Ref: 12888923 Int J Oncol, 2003
- Furthermore, NAMI A through modulation of PKC activity has been proved capable of reducing the phorbol ester induced expression of ornithine decarboxilase ODC gene and to abrogate the activation of the Raf  [ **RAF1** ]  MEK  [ **MAP2K1** ]  ERK pathway.  Ref: 12851698 Int J Oncol, 2003
- Our aim was to determine whether activation of the raf 1  [ **RAF1** ]  MEK  [ **MAP2K1** ]  MAPK signal transduction pathway in carcinoid cells could modulate production of neuroendocrine markers and hormones.  Ref: 12851216 Am J Physiol Gastrointest Liver Physiol, 2003
- Taken together, our results identify a novel mechanism whereby a Gi o protein coupled receptor inhibits cell proliferation by stimulating ERK signaling via a SHP 1 SHP 2 PI3K Ras Rap1 B Raf  [ **RAF1** ]  MEK  [ **MAP2K1** ]  pathway.  Ref: 12878607 J Biol ChemJ Biol Chem, 2003
- Gastrin activated transcription through a Raf  [ **RAF1** ] , Mek  [ **MAP2K1** ]  and Erk dependent but Ras independent pathway.  Ref: 12891554 Gastroenterology, 2003
